# Characterization of Interictal Epileptiform Discharges with Time-Resolved Cortical Current Maps Using the Helmholtz–Hodge Decomposition

**DOI:** 10.3389/fneur.2012.00138

**Published:** 2012-10-10

**Authors:** Jeremy D. Slater, Sheraz Khan, Zhimin Li, Eduardo Castillo

**Affiliations:** ^1^Department of Neurology, University of Texas Health Science Center at HoustonHouston, TX, USA; ^2^Department of Neurology, Massachusetts General Hospital, University of Texas Health Science Center at HoustonHouston, TX, USA; ^3^Department of Pediatrics, University of Texas Health Science Center at HoustonHouston, TX, USA; ^4^Pediatric Epilepsy Program, Florida Hospital in OrlandoOrlando, FL, USA

**Keywords:** epilepsy, epileptic spike localization, magnetoencephalography, optical flow, dipole modeling

## Abstract

Source estimates performed using a single equivalent current dipole (ECD) model for interictal epileptiform discharges (IEDs) which appear unifocal have proven highly accurate in neocortical epilepsies, falling within millimeters of that demonstrated by electrocorticography. Despite this success, the single ECD solution is limited, best describing sources which are temporally stable. Adapted from the field of optics, optical flow analysis of distributed source models of MEG or EEG data has been proposed as a means to estimate the current motion field of cortical activity, or “cortical flow.” The motion field so defined can be used to identify dynamic features of interest such as patterns of directional flow, current sources, and sinks. The Helmholtz–Hodge Decomposition (HHD) is a technique frequently applied in fluid dynamics to separate a flow pattern into three components: (1) a non-rotational scalar potential **U** describing sinks and sources, (2) a non-diverging scalar potential **A** accounting for vortices, and (3) an harmonic vector field **H**. As IEDs seem likely to represent periods of highly correlated directional flow of cortical currents, the **U** component of the HHD suggests itself as a way to characterize spikes in terms of current sources and sinks. In a series of patients with refractory epilepsy who were studied with magnetoencephalography as part of their evaluation for possible resective surgery, spike localization with ECD was compared to HHD applied to an optical flow analysis of the same spike. Reasonable anatomic correlation between the two techniques was seen in the majority of patients, suggesting that this method may offer an additional means of characterization of epileptic discharges.

## Introduction

Source localization of interictal epileptiform discharges (IEDs) recorded with electroencephalography has traditionally been performed via visual analysis. As Rose and Ebersole have written, visual analysis relies on three assumptions: (1) the electrode(s) demonstrating the highest amplitude abnormal potential directly overlie the generator, (2) a cortical generator always produces a focal potential, and (3) a widespread potential indicates a diffuse source or multiple sources (Rose and Ebersole, 2009). All three assumptions are frequently incorrect. To go beyond visual analysis, a variety of mathematical techniques of source localization have been proposed and utilized including equivalent current dipoles (ECD; Henderson et al., 1975), minimum norm estimates (MNE; Hauk, 2004; Silva et al., 2004), standardized low-resolution brain electromagnetic tomography (sLORETA; Pascual-Marqui, 2002), and dynamic statistical parametric mapping (dSPM; Tanaka et al., 2009). Each of these techniques have advantages and disadvantages, but particularly in combination with higher density EEG arrays and magnetoencephalography, the single ECD model has proven value in helping to identify the ictal onset zone in neocortical epilepsies (Huiskamp et al., 2010; Shiraishi, 2011). As an example, source estimates performed using a single ECD model for IEDs which appear predominantly unifocal in their generation such estimates have proven to be highly accurate, falling within millimeters of those demonstrated by electrocorticography (Ishibashi et al., 2002). Despite this success, the single ECD solution is limited, best describing non-moving sources. Sources that move over time have been described by moving dipoles or multiple dipole models (Ochi et al., 2000), or iterative application of the ECD model (Papanicolaou et al., 2006), but mathematical solutions that incorporate more details of the complexity of the generating cortical tissue have multiple solutions, become computationally intractable, or both (Yetik et al., 2005). Alternative methods for IED source localization such as minimum norm estimation have been proposed, and may be theoretically better than ECD given the potential complexity of the generators involved, but the vast majority of clinical validation has been performed with the ECD model (Wheless et al., 1999; Pataraia et al., 2004). Given the fairly large area of cortex involved in spike generation (at least those identified on scalp EEG which serve as the basis for MEG ECD localization), the limitation of the ECD method in reducing cortical sources to dimensionless points renders it less than ideal. While appropriate pre-processing of the signal data and adjustments to the head model can produce significant improvements in source localization results, all of these methods generally evaluate a single point in time, so that a spike, for example, is reduced to the moment of peak negativity (unless you use the ECD iteratively). An analysis method that evaluates signal change over time might contribute useful information to the existing models.

In the field of optics, the goal of optical flow estimation is to compute an approximation to the motion field from time-varying image intensity (Fleet and Weiss, 2005). Optical flow analysis of distributed source models of MEG or EEG data has recently been proposed as a means to estimate the current motion field of cortical activity, or “cortical flow.” This technique can be used to estimate local kinetic energy of cortical surface currents, and has been used to characterize correspondence between the speed and direction of the surface current flow within the visual cortex and the dynamical properties of the visual stimulus itself (Lefevre and Baillet, 2008, 2009). The motion field so defined can be used to identify dynamic features of interest such as patterns of directional flow, current sources, and sinks.

The Helmholtz–Hodge Decomposition (HHD) is a technique frequently applied in fluid dynamics to separate a flow pattern into three components: (1) a non-rotational scalar potential ***U*** describing sinks and sources, (2) a non-diverging scalar potential ***A*** accounting for vortices, and (3) an harmonic vector field ***H*** (Chorin and Marsden, 1993; Tong et al., 2003). A recently published abstract demonstrated the use of the HHD for mapping and characterizing current flow over primary somatosensory cortex during sensory-stimulation triggered evoked potentials (Khan et al., 2009). As IEDs seem likely to represent periods of highly correlated directional flow of cortical currents, the HHD ***U*** potential lends itself to the characterization of spikes in terms of current sources and sinks. The methodology is reviewed in greater detail in the addendum.

In this study, its relative efficacy compared to that of the standard ECD model, was assessed with a series of six candidates for epilepsy surgery.

## Materials and Methods

### MEG recording

All patients underwent an MEG recording sessions. MEG recordings were performed pre-operatively for localization of the sources of interictal epileptiform activity. Spontaneous MEG was recorded with a whole-head neuromagnetometer containing 248 first-order axial gradiometer channels (Magnes WH3600, 4-D Neuroimaging, San Diego, CA, USA) in a magnetically shielded room. Simultaneous EEG was recorded, with gold disk electrodes, using a bipolar montage (Neurofax, Nihon-Kohden, Tokyo, Japan) from 21 scalp locations, placed according to the International 10–20 system. The MEG recordings were digitized at a sampling rate of 508.63 Hz. The online bandpass filter was set between 1 and 200 Hz.

As part of the analysis of the MEG-recorded interictal paroxysmal activity, we calculated the ECD location, orientation, and moment for each event. Concurrently recorded EEG was used to identify interictal epileptiform event sand to rule out artifacts, such as those produced by body or eye movements, cardiac, and sleep-related activity. We used single epileptiform events for source localization in order to avoid introducing artificial time delays by averaging variable spike populations. Calculation of the location, orientation, and strength of the dipolar sources that best fitted the measured magnetic fields was performed using the single, moving, ECD model that is part of the 4-D Neuroimaging software. The algorithm was applied to magnetic flux distributions that showed clear and stable dipolar morphology. For each calculation, magnetic flux data from 37 magnetometer sensors were used, encompassing both extrema of the dipolar surface distribution. For each epileptiform event source solutions were examined every 2 ms during a 200-ms window (100 ms before and 100 ms after the peak of the interictal spike complex). The goal of this method was to find the best combination of ECD location, strength, and orientation parameters. A dipole solution was considered acceptable if it was associated with a correlation coefficient of 0.95 or greater, global field power (GFP; or root mean square of the magnetic flux in the set of 37 magnetometer sensors entered in the analysis) of 400 ft or greater, and an ECD product moment of 400 nAm or less. The methodology is the same as that used in a prior report from our group (Pataraia et al., 2004).

For the purpose of identifying the location of the estimated sources in the brain, an magnetic resonance imaging (MRI) scan was performed. Before scanning, three skin markers were placed at fiducial points on the patient’s head (the nasion, the left, and the right external meati). The location of the same fiducial points was also recorded, at the beginning of the MEG recording session, relative to the MEG sensor, thus establishing a common spatial reference for the transposition of 3-D coordinates between MEG and MRI data, as previously described.

### Cortical surface reconstruction

Cortical surface segmentation and tessellation from T1-weighted axial MRI scans (1 mm× 1 mm× 1 mm^3^ voxel size) was obtained using BrainSuite software (Shattuck and Leahy, 2002)^1^. Data analysis was performed with Brainstorm (Tadel et al., 2011), which is documented and freely available for download online under the GNU general public license^2^.

Three-dimensional reconstruction of the head and cortical surface was carried out for each patient individually. For forward modeling of MEG signals, an overlapping spheres head model was computed using the method of Huang et al. (1999). The computed head and cortex models were used in combination with the MEG fields to compute an estimate of current-source density distribution over the cortex based on a Tikhonov-regularized minimum norm estimate (Baillet et al., 2001). The default value for the Tikhonov parameter is λ = 10% of maximum singular value of the lead field.

### Optical flow and HHD

Optical flow and HHD data analysis were also performed with Brainstorm (Tadel et al., 2011). Pre-processing of the MEG data consisted of applying a low-pass filter of 30 Hz to minimize distortions produced by higher frequency jitter. The computed head and cortex models were used in combination with the MEG fields to compute an estimate of the current-source density distribution over the cortex based on a minimum norm estimate. Optical flow velocity fields were computed from the cortical current distribution estimated over the individual cortical surface of each subject. For each spike identified, the MEG recording for a time period covering spike onset, peak, and offset (as identified on the corresponding scalp EEG) was subjected to optical flow analysis, and subsequent HHD. The GFP was compared to the global dynamic energy (DE) measurement.

$$\text{DE}\left(t\right)=\int_{M}{\left\|v\right\|}^{2}\text{d}\mu$$

DE = displacement energy
**V** = vector field
*M* = surface manifold (in this case, the cortex)

The peak DE occurring prior to the peak GFP during a spike identified the time of the source. The peak DE occurring after the peak GFP during the same spike identified the sink. The HHD ***U*** potential was then calculated for the source time point and sink time point and plotted over the cortical manifold.

As this method results in a broad area of simulated current flow, rather than a focal point, localization was based on the location of the visible maximum in terms of lateralization and lobe. This localization was then classified as either concordant or discordant with the dipole calculated for the same spike.

## Results

**Figure 1:**
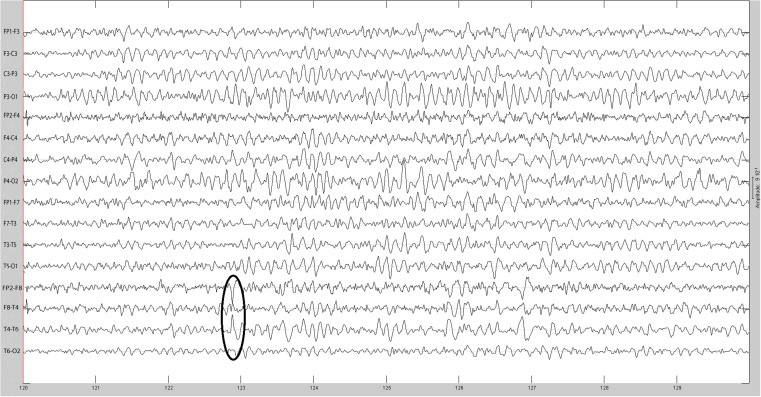
**Original spike at time 122.8732 s**.

**Figure 2:**
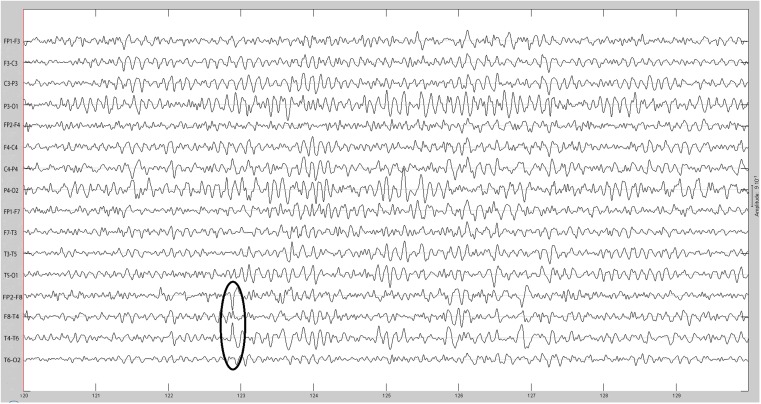
**Same spike as Figure 1 after 30 Hz low-pass filtering**.

**Figure 3:**
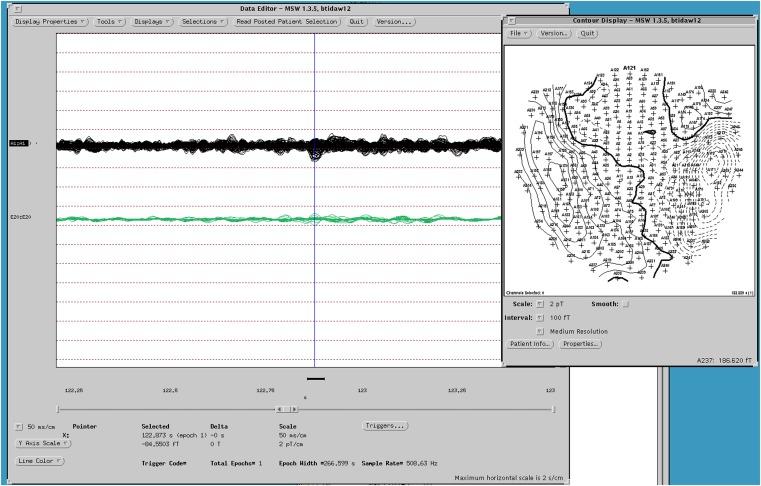
**MEG dipole map of spike from Figure 1**.

**Figure 4:**
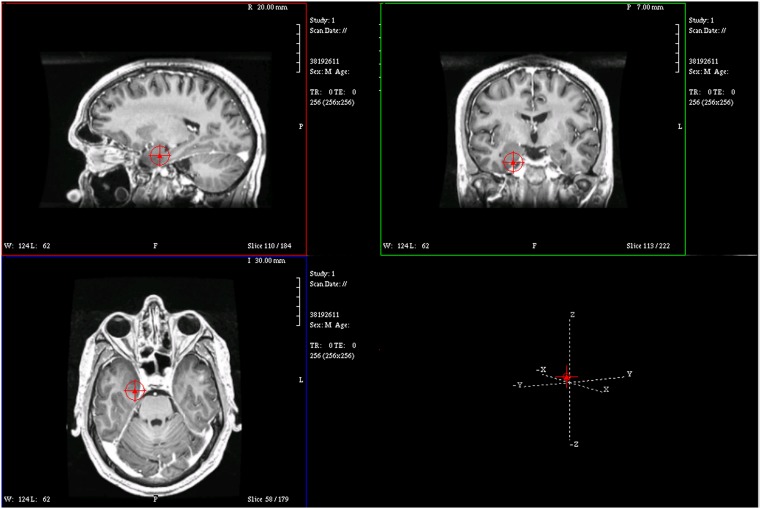
**Magnetic resonance imaging plot of ECD of spike from Figure 1**.

**Figure 5:**
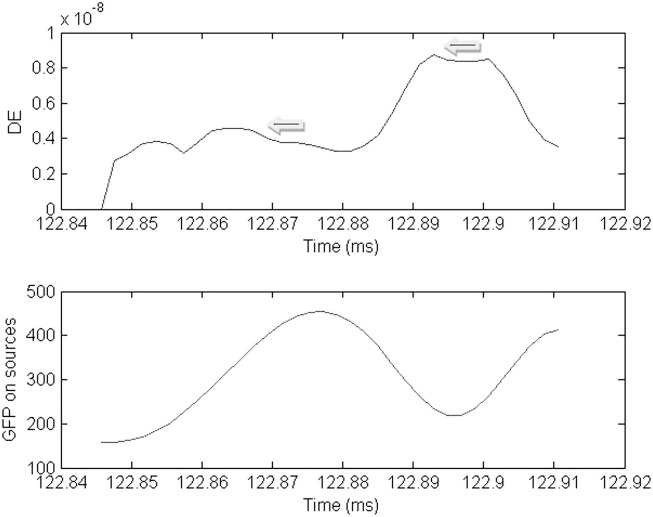
**Plot of the dynamic energy (DE) and global field power (GFP) over the time course of the spike from Figure 1**. Arrows mark the DE maxima before and after the spike peak (which in this instance corresponds to the GFP maximum).

The sequential results for the first patient (2092) are presented in detail. The original spike prior to filtering is shown in Figure 1. The same spike after 30 Hz low-pass filtering is illustrated in Figure 2. The same discharge in the context of alpha background rhythm demonstrating a right temporal preponderance on MEG is shown in Figure 3. The MEG demonstrated only a weak dipolar map over right temporal area. The plot of the calculated ECD on the patient’s MRI, revealing a right mesial temporal localization, is shown in Figure 4. The DE map, with the source and sink time points marked, is shown in Figure 5.

**Figure 6:**
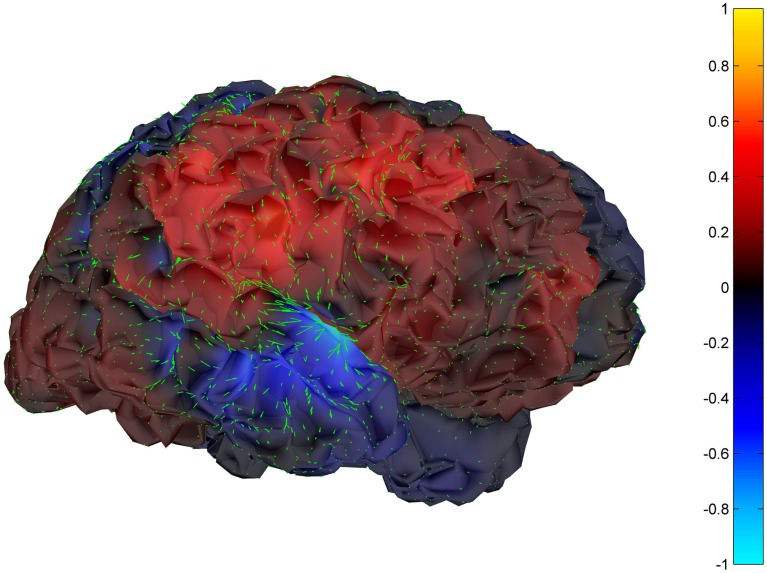
**Helmholtz–Hodge decomposition source field plot of the spike from Figure 1 (at time = 122.8653 s)**. The color bar corresponds to the normalized amplitude of the component of current flow perpendicular to the cortical manifold, positive if directed inward (red = sink), negative if outward (blue = source).

**Figure 7:**
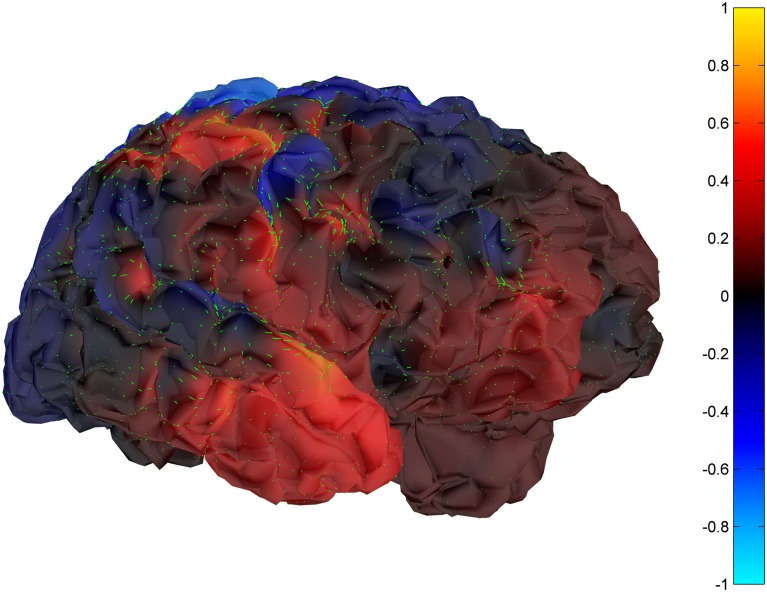
**Helmholtz–Hodge decomposition sink field plot of the spike from Figure 1 (at time = 122.8929 s)**. The color bar corresponds to the normalized amplitude of the component of current flow perpendicular to the cortical manifold, positive if directed inward (red = sink), negative if outward (blue = source).

Cortical surface localization of spike source and sink are shown on Figures 6 and 7 respectively, where blue shading of the cortical manifold indicates the outward current flow or source, and red shading indicates the inward current flow or sink. For those figures, the arrow size in each region gives an approximation of relative current magnitude.

**Figure 8:**
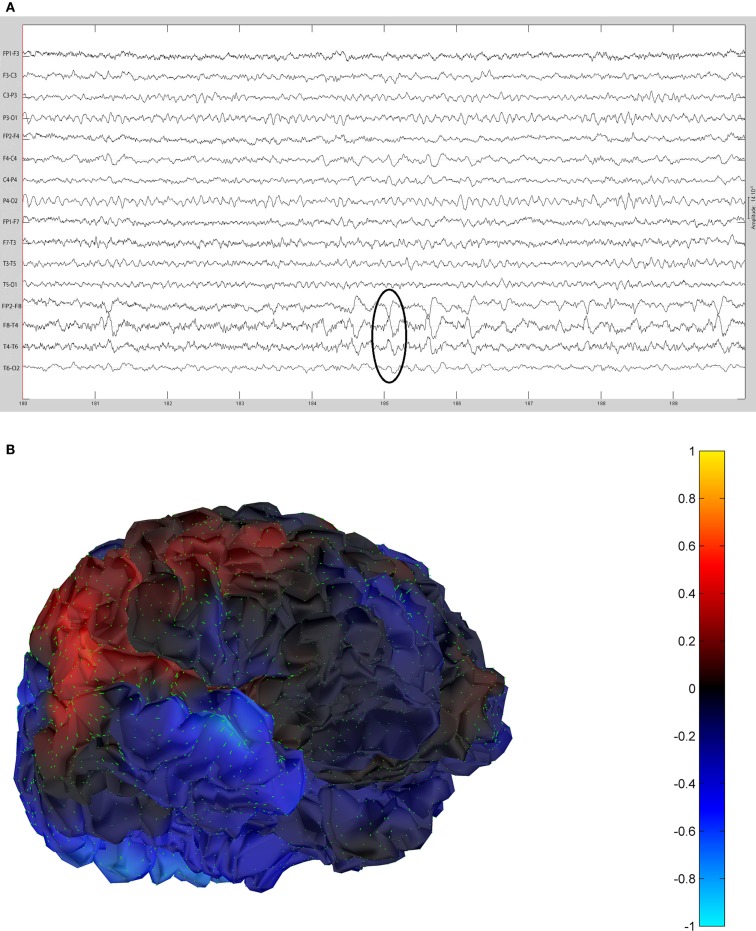

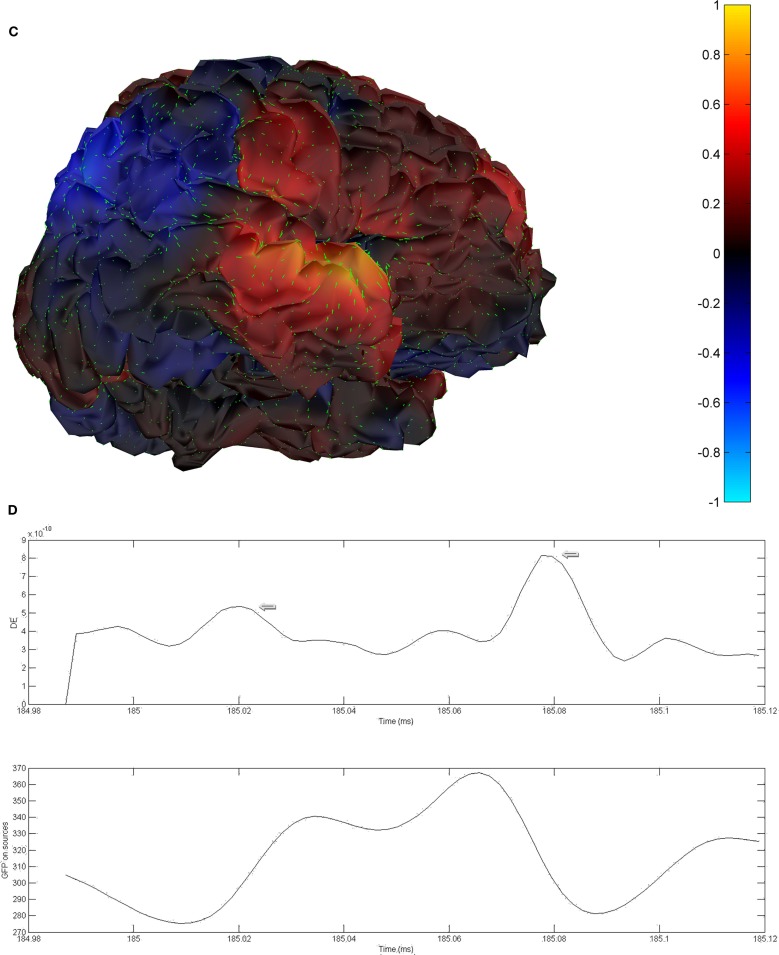
**(A)** Sample sharp wave from patient 2243 (highlighted with black oval). **(B)** HHD source field plot of the spike from patient 2243. **(C)** HHD sink field plot of the spike from patient 2243. **(D)** Plot of the dynamic energy (DE) and global field power (GFP) over the time course of the spike from patient 2243. Arrows mark the DE maxima before and after the spike peak.

**Figure 9:**
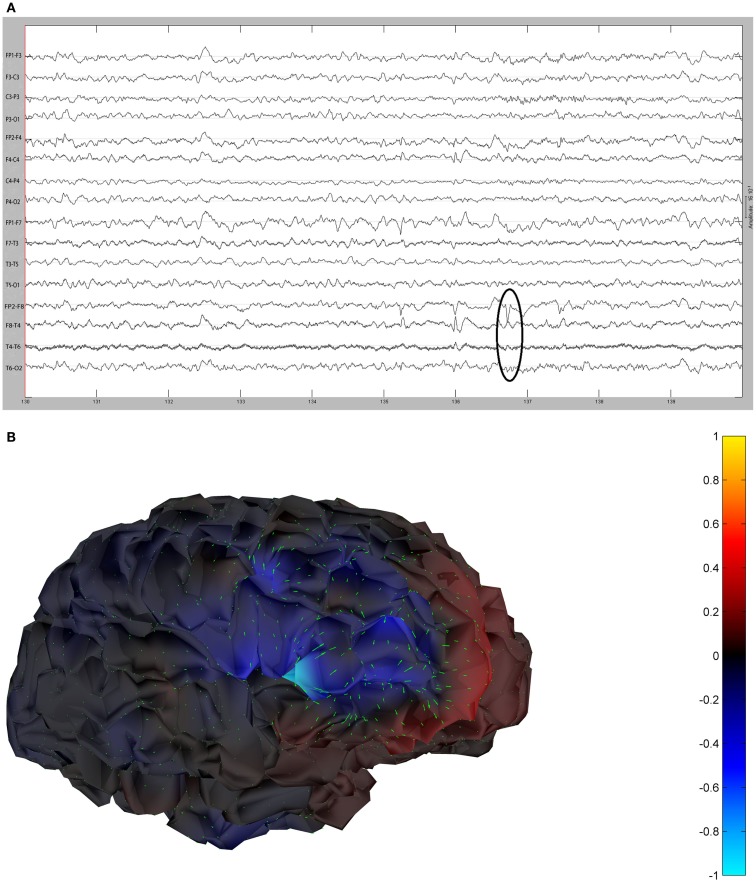

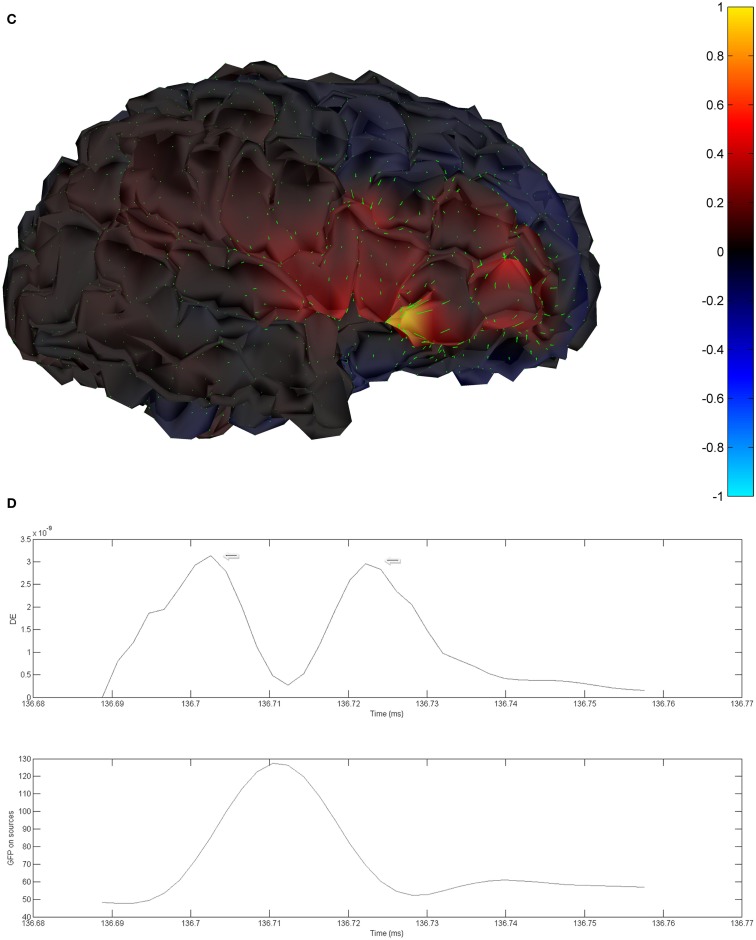
**(A)** Sample sharp wave from patient 2296 (highlighted with black oval). **(B)** HHD source field plot of the spike from patient 2296. **(C)** HHD sink field plot of the spike from patient 2296. **(D)** Plot of the dynamic energy (DE) and global field power (GFP) over the time course of the spike from patient 2296. Arrows mark the DE maxima before and after the spike peak.

**Figure 10:**
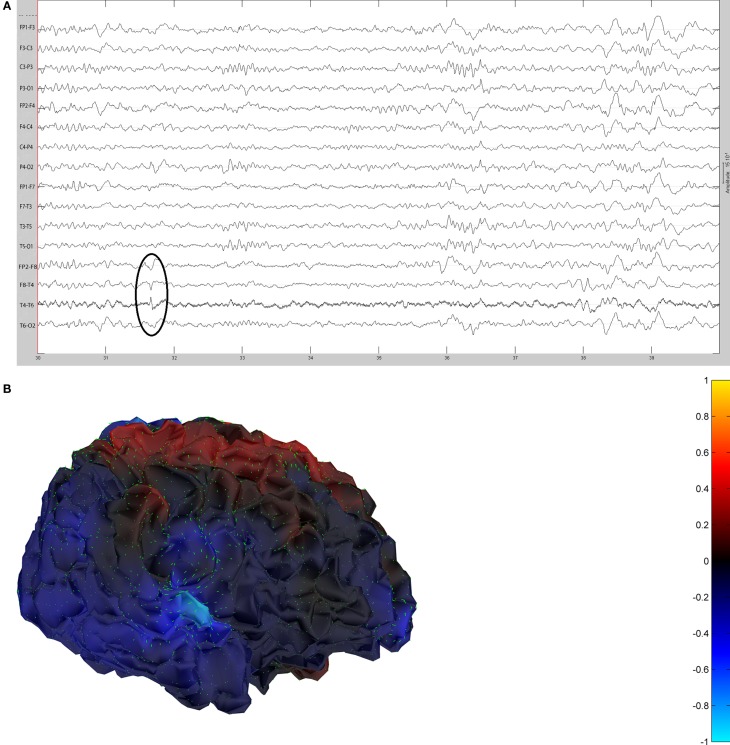

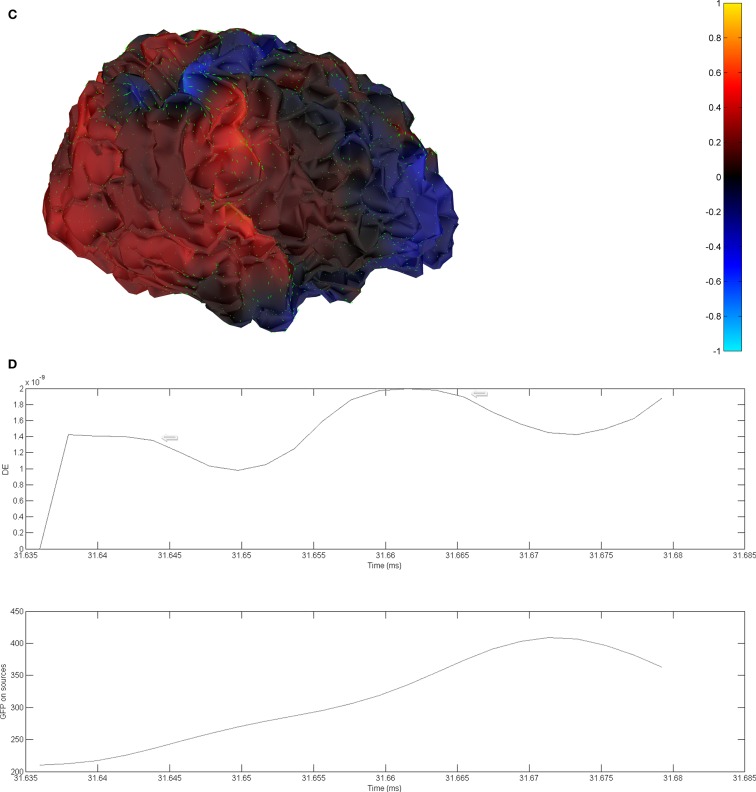
**(A)** Sample sharp wave from patient 2334 (highlighted with black oval). **(B)** HHD source field plot of the spike from patient 2334. **(C)** HHD sink field plot of the spike from patient 2334. **(D)** Plot of the dynamic energy (DE) and global field power (GFP) over the time course of the spike from patient 2334. Arrows mark the DE maxima before and after the spike peak.

**Figure 11:**
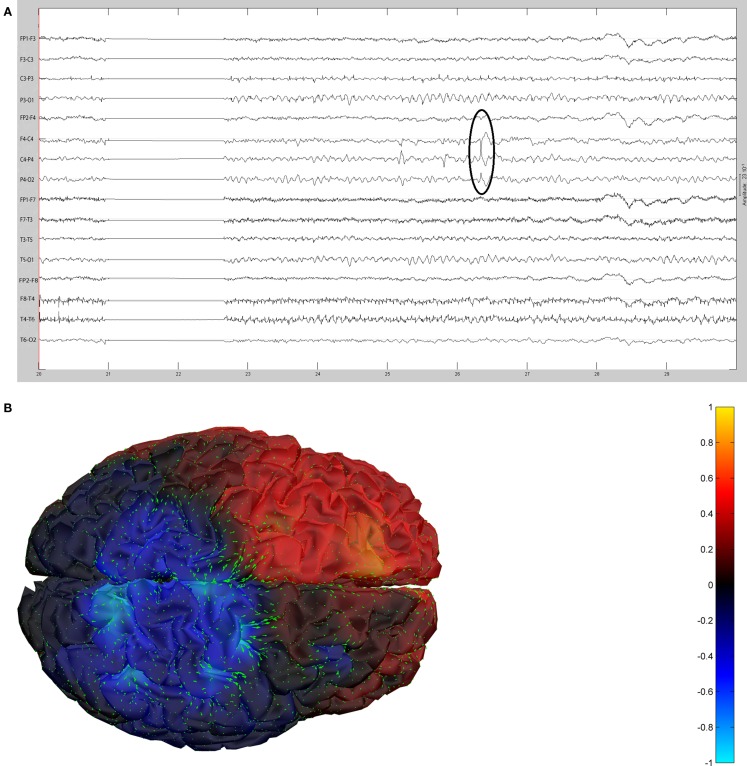

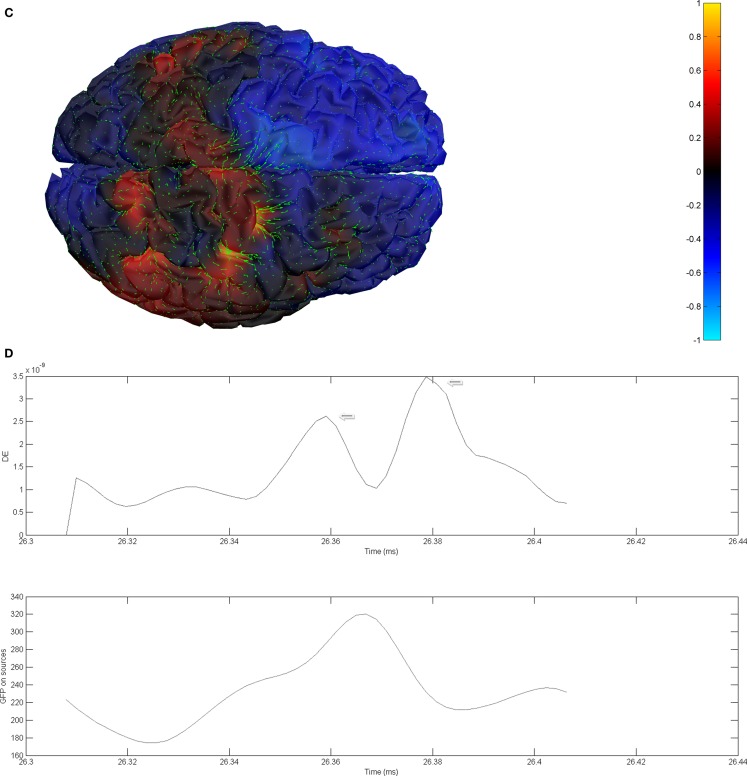
**(A)** Sample sharp wave from patient 2353 (highlighted with black oval). **(B)** HHD source field plot of the spike from patient 2353. **(C)** HHD sink field plot of the spike from patient 2353. **(D)** Plot of the dynamic energy (DE) and global field power (GFP) over the time course of the spike from patient 2353. Arrows mark the DE maxima before and after the spike peak.

**Figure 12:**
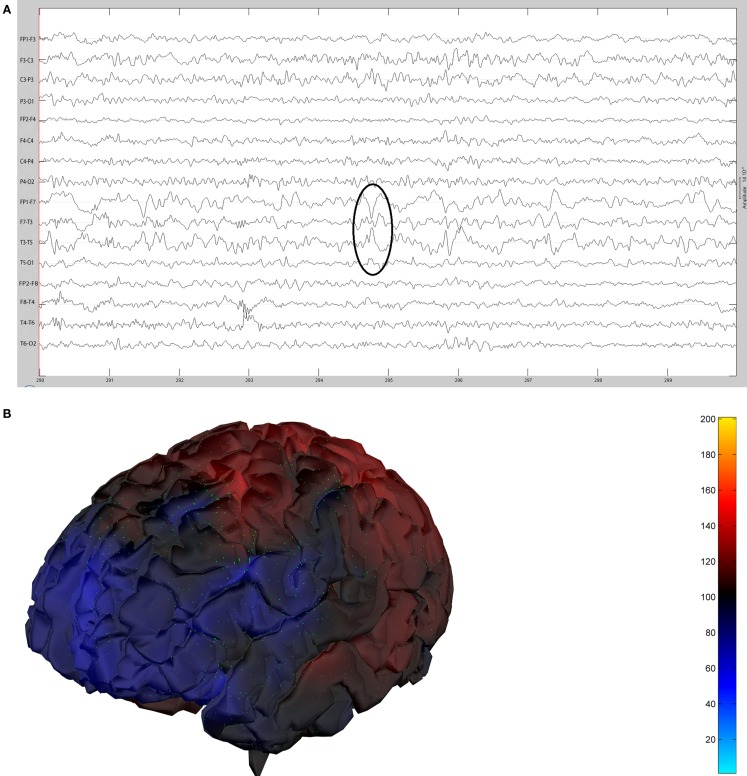

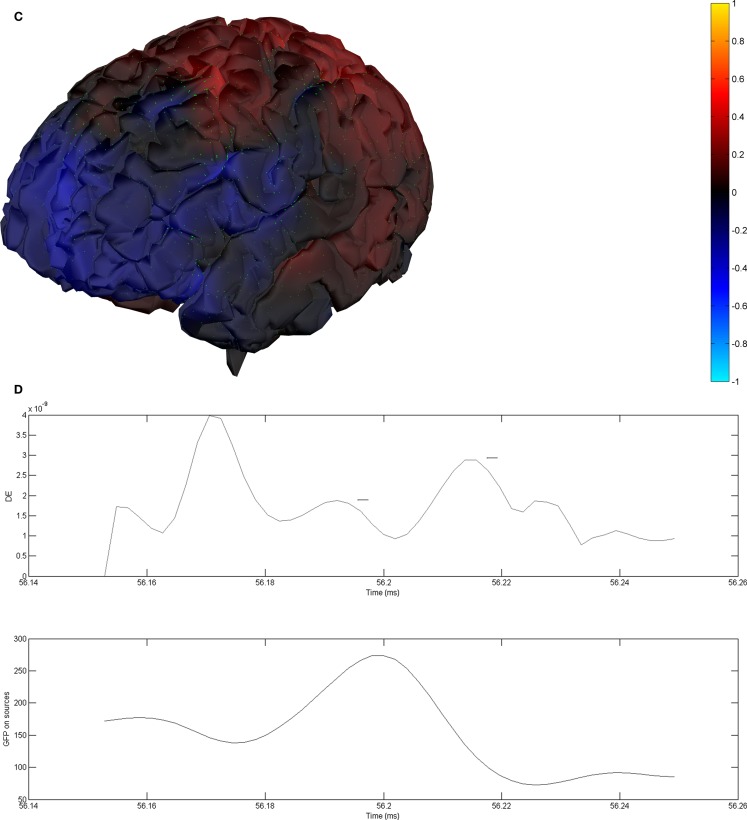
**(A)** Sample sharp wave from patient 2406 (highlighted with black oval). **(B)** HHD source field plot of the spike from patient 2406. **(C)** HHD sink field plot of the spike from patient 2406. **(D)** Plot of the dynamic energy (DE) and global field power (GFP) over the time course of the spike from patient 2406. Arrows mark the DE maxima before and after the spike peak.

The remaining five patients are summarized in Figures 8–12. The first Figure in each set (a) shows the original spike or sharp wave as identified on scalp EEG. For each patient, the best example of a given spike population has been chosen for presentation. The second figure (b) is the HHD source map. The third figure (c) is the HHD sink map. The fourth figure (d) is the combined plot of the DE and GFP.

Table 1 lists the six patients whose interictal activity was analyzed, including: the dipole localization using ECD of most of IEDs recorded during the session, whether or not the HHD topographic plot was concordant with the ECD localization and the pathologic diagnosis if known. For five out of six subjects, the HHD projection was concordant with the ECD localization. With the sixth, the ECD localization was in the left perisylvian region, concordant with the pathology visible on MRI, but the HHD sink and source, while broad and low amplitude, were maximal over the lateral frontal lobe.

**Table 1:** **MEG dipole localization, concordance and diagnosis**.

ID	MEG dipole localization	HHD/optical flow analysis concordant with dipole analysis	Diagnosis (if known)
2092	Right temporal	Yes	Right mesial temporal sclerosis (surgical pathology)
2243	Right mesial temporal	Yes	Right mesial temporal sclerosis (surgical pathology)
2296	Right frontal	Yes	Right mesial temporal sclerosis (by MRI, no pathology available)
2234	Right temporal	Yes	Right mesial temporal sclerosis (surgical pathology)
2353	Right parietal	Yes	Right parietal low grade glioma (surgical pathology)
2406	Left temporal (perisylvian)	No (left lateral frontal)	Abnormal signal and architectural abnormality in the tail of the left hippocampus on MRI

## Discussion

For most cases, spikes and sharp waves recorded with simultaneous EEG/MEG, the source and sink areas defined by HHD of the optical flow analysis of the MEG signals appear concordant with the location identified by ECD. Where the two techniques are in disagreement, the divergence may be due to a failure of the OF/HHD to make an accurate localization, or it may be due to an error on the part of the ECD localization. An additional possibility is that neither is in error, but rather the area of cortex identified by each model is different simply because the models represent alternative aspects of the biomagnetic activity (a point dipole is not a current flow). The OF analysis is based on the minimum norm estimate source modeling of the magnetic activity, and so is subject to the limitations of that model. By its nature, OF/HHD describes a region, not a point, and given that the underlying cortical activation generating a discharge visible on a scalp recording is virtually never a point, this may be a more intuitive representation. Compared to the minimum norm estimate, the OF/HHD analysis may have an advantage in the defined direction of current flow, which not only allows for tracking the region of involvement over time, but the characterization of regions of cortex as source and sink for the discharges. At a minimum, this allows for differentiation of stationary versus “moving” sharp waves. Whether the area of involvement reflects source depth or the extent of the epileptic zone remains speculative at this time. As might be expected, the point in time of the traditional spike “peak” frequently occurs at a point of maximal GFP and minimal DE. This was less consistent when other high energy and or high frequency activity occurred concurrent with the spike generation, and a more accurate depiction might result from limiting the analysis to a region of interest, rather than the entire cortical surface.

One limitation of the current study is the relatively small number of patients included in the analysis. While any number of factors might prevent a subject from inclusion during the period of data collection, the most common reason was that no interictal discharges were recorded during the MEG session. A second limitation is the lack of medial cortical surface views, particularly critical for patients with mesial temporal discharges. In the instance of subject 2406, where the HHD localization was discordant from that of the ECD, the absolute amplitude of the source and sink flow vectors are less than maximal. As the magnitude of the flow vectors are normalized for each HHD image, this implies that there sink and source regions of greater amplitude that are not visible with the available set of views. The software used for the current analysis lacks the capacity to present these views, but one may anticipate that increased use in the community of the HHD analysis component of the software will result in this functionality being added.

Many other questions remain to be answered. Defining the relationship (if any) between the source/sink areas and the epileptogenic zone is important, but of greater importance is discovering if OF/HHD can be used in relationship to the eventual surgical resection to predict outcome. Association of particular source/sink patterns with specific pathology should also be investigated, in addition to the potential effects of drugs such as antiepileptic medications.

This study represents the first use of OF/HHD techniques that we are aware of for the localization of epileptic activity. While this approach is novel, it is important to emphasize that despite the appearance of the graphics and the terms source and sink giving the appearance of electrical currents over the cortex, it seems exceedingly unlikely that the actual surface dynamics of the electrical activity of the cortex resemble that which is depicted. The MEG spike data suffers from the same limitations that all MEG recordings do, namely that the MEG is relatively insensitive to superficial radial sources (Nunez and Srinivasan, 2006). While the argument has been made that use of the MNE is the optimal solution for the inverse problem of bioelectromagnetic source localization (Hauk, 2004), the model remains limited by the data upon which it is based. Thus the eventual value of the OF/HHD analysis of spikes will be only determined by the degree to which it demonstrates clinically useful correlations with pathologic brain states.

## Conflict of Interest Statement

The authors declare that the research was conducted in the absence of any commercial or financial relationships that could be construed as a potential conflict of interest.

## Figures and Tables

**Figure A1:**
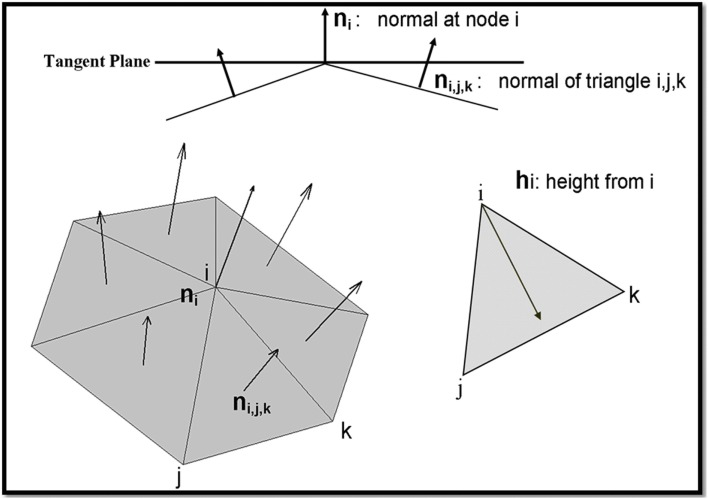
**Construction of the manifold approximation from the composite nodes and triangles**.

**Figure A2:**
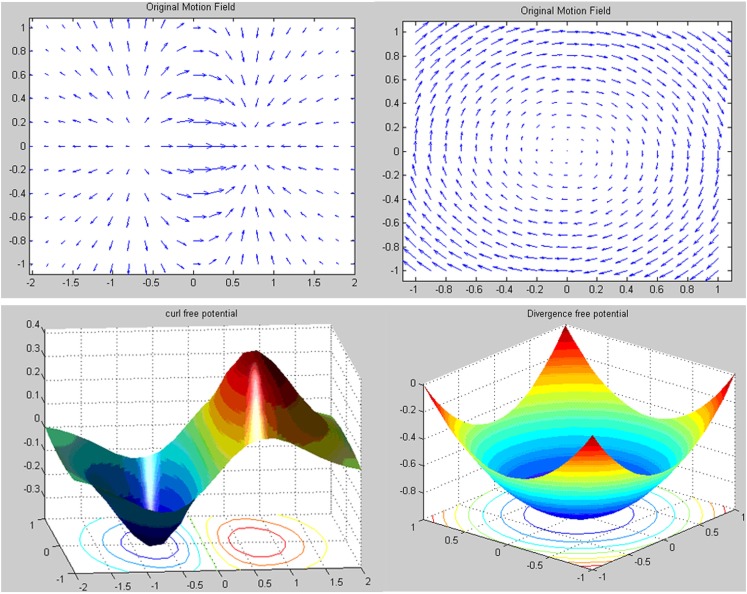
**Illustration of two motion fields, one purely demonstrating the curl-free activity and the second the divergence-free activity**. The top two illustrations are the original motion fields. The bottom two are the corresponding plots of the U and A fields.

## Footnotes

^1^http://www.loni.ucla.edu/Software/BrainSuite

^2^http://neuroimage.usc.edu/brainstorm

## Appendix

### Optical flow and HHD theory review

The critical assumption for use of optical flow to approximate cortical surface current flow is a conservation of intensity:

$$\begin{array}{c} I\left(x,t\right)=I\left(x+dx,t+dt\right)=I\left(x,t\right)+\frac{\partial I}{\partial x_{1}}dx_{1}+\frac{\partial I}{\partial x_{2}}dx_{2}+\frac{\partial I}{\partial t}dt+\ldots \end{array}$$

While the concept that the overall energy level at the cortical surface remains constant over time is clearly incorrect, the assumption as an approximation is reasonable for a small enough time interval compared to the time scale of the phenomenon being evaluated.

The first-order approximation:

$$\frac{\partial I}{\partial x_{1}}\frac{dx_{1}}{dt}+\frac{\partial I}{\partial x_{2}}\frac{dx_{2}}{dt}+\frac{\partial I}{\partial t}=0$$

leading to the constraining equation for optical flow:

$$\frac{\partial I}{\partial t}+\nabla I\cdot V=0$$

For a two-dimensional Riemannian manifold:

$$\partial_{t}I+V\cdot \nabla_{M}I=0$$

The Helmholtz–Hodge decomposition (HHD) is a method to detect features in vector field. Borrowing from the ideas of fluid dynamics, identifiable features of current flow fields include sources, sinks, and vortices. For purposes of characterizing epileptic spikes, the identification of the portion of current flow that emerges perpendicular to the cortical surface (the source) and conversely the portion of current flow directed inward, again perpendicular to the surface (the sink) suggest themselves as potentially important features to extract from the overall flow pattern.

For a vector field **V**, there exist two potentials U and **A** and a vector field h such that

V=∇U+∇×A+H

where U is a scalar potential and A is a vector potential. To ensure the uniqueness of the decomposition, boundary conditions have to be introduced: scalar potential U is demanded to be normal to the boundary while curl A has to be tangential to it.

$$\text{V}=\nabla_{M}U+\text{Cur}{\text{l}}_{M}A+H$$

With the following properties:

$$\begin{array}{rrrr} \text{Cur}{\text{l}}_{M}\left(\nabla_{M}U\right)=0, & & & \\ \text{di}{\text{v}}_{M}\left(\text{Cur}{\text{l}}_{M}A\right)=0, & & & \\ \text{di}{\text{v}}_{M}H=0, & & & \\ \text{Cur}{\text{l}}_{M}H=0. & & & \end{array}$$

U and A minimize the two functionals:

$$\begin{array}{rcll} \int_{M}{∥V-\nabla_{M}U∥}^{2} & & & \\ \int_{M}{∥V-\text{Cur}{\text{l}}_{M}A∥}^{2} & & & \end{array}$$

where ||.|| is the norm associated with the Riemannian metric *g*(.,.). These two functionals are convex therefore they have unique minimum on ***L^2^(M)*** which satisfies:

$$\begin{array}{rcll} \forall \phi \in L^{2}\left(M\right),\int_{M}g\left(V,\nabla_{M}\phi \right) & =\int_{M}g\left(\nabla_{M}U\text{,}\nabla_{M}\phi \right) & & \\ \forall \phi \in L^{2}\left(M\right),\int_{M}g\left(V,\text{Cur}{\text{l}}_{M}\phi \right) & =\int_{M}g\left(\text{Cur}{\text{l}}_{M}A,\text{Cur}{\text{l}}_{M}\phi \right) & & \end{array}$$

If we have basis functions (ϕ_1_, …, ϕ*_n_*), then we can write **U** = (*U*_1_, …, *U_n_*)*^T^*, **A** = (*A*_1_, …, *A_n_*)*^T^*.

$${\left[\int_{M}g\left(\nabla_{M}\phi_{i},\nabla_{M}\phi_{j}\right)\right]}_{i,j}U={\left[\int_{M}g\left(V,\nabla_{M}\phi_{i}\right)\right]}_{i}$$

$${\left[\int_{M}g\left(\text{Cur}{\text{l}}_{M}\phi_{i},\text{Cur}{\text{l}}_{M}\phi_{j}\right)\right]}_{i,j}A={\left[\int_{M}g\left(V,\text{Cur}{\text{l}}_{M}\phi_{i}\right)\right]}_{i}$$

with tessellation ℳ approximating the manifold consisting of *N* nodes and *T* triangles (Figure A1).

$$\left[\underset{T∍i,j}{\sum }\frac{h_{i}}{{∥h_{i}∥}^{2}}\cdot \frac{h_{j}}{{∥h_{j}∥}^{2}}A\left(T\right)\right]U=\left[\underset{T∍i}{\sum }A\left(T\right)V\cdot \frac{h_{i}}{{∥h_{i}∥}^{2}}\right]$$

$$\begin{array}{c} \left[\underset{T∍i,j}{\sum }\left(\frac{h_{i}}{{∥h_{i}∥}^{2}}\wedge n\right)\cdot \left(\frac{h_{j}}{{∥h_{j}∥}^{2}}\wedge n\right)A\left(T\right)\right]A=\left[\underset{T∍i}{\sum }A\left(T\right)V\cdot \left(\frac{h_{i}}{{∥h_{i}∥}^{2}}\wedge n\right)\right] \end{array}$$

Where **h***_i_* is the height taken from the *i* in the triangle *T*. ((*T*) is the area of the triangle *T*. **n** is the normal to the triangle *T*.

The result is that *U* is a curl-free potential and *A* is a divergence free potential (Figure A2).
